# Morphometric Study of Mitral Valve Annulus in Iran

**DOI:** 10.22086/gmj.v0i0.1078

**Published:** 2018-03-31

**Authors:** Tahereh Mohtaj, Mathias H. Aazami, Ghassem Sazegar, Hoorak Poorzand, Aria Hedjazi, Negar Morovatdar, Ahmad Toorabi

**Affiliations:** ^1^Department of Cell Biology and Anatomy, School of Medicine, Medical Sciences University, Mashhad, Iran; ^2^Javad Al-Aemeh Heart Hospital, Honarestan Avenue, Mashhad, Iran; ^3^Cardiac Anesthesia Research Center, Mashhad University of Medical Sciences, Imam Reza Teaching Hospital, Ibn-Sina Avenue, Mashhad, Iran; ^4^Macroanatomy Research Center, Mashhad University of Medical Sciences, Mashhad, Iran; ^5^Atherosclerosis Prevention Research Center, Department of Cardiology, Imam Reza Hospital, Faculty of Medicine, Mashhad University of Medical Sciences, Mashhad, Iran; ^6^Legal Medicine Research Center, legal Medicine Organization, Tehran, Iran; ^7^Clinical Research Unit, Faculty of Medicine, Mashhad University of Medical Sciences, Mashhad, Iran; ^8^Deputy of Culture and Ethics, School of Medicine, Mashhad University of Medical Sciences, Vakil Abad Avenue, Mashhad, Iran

**Keywords:** Mitral Valve, Cadaver, Cardiac Morphology

## Abstract

**Background:**

This study aimed to determine the normal dimensions of the mitral annulus (MA) in Iranian population.

**Materials and Methods:**

This cross-sectional study was conducted using 88 fresh hearts of male and female cadavers for six months in Mashhad, Iran. Normal data were determined by measuring the exact dimensions of the MA in fresh hearts of patients who had died of non-cardiac causes and considering some parameters such as age, gender, stature, and weight. Images of the valves and leaflets were prepared by marking the anterior (A2, midpoint of anterior) and posterior areas of P1, P2, and P3 using a needle. To analyze the data, SPSS version 16 was used.

**Results:**

The means of anatomic area, anatomic perimeter, inter-commissural distance, A2-P1, A2-P2, A2-P3, Base-P1, Base-P2, Base-P3, and Base-A were 14±1.28, 8.3±1, 2.7±0.42, 2.27±0.37, 2.3±0.43, 2.06±0.35, 1.66±0.43, 1.2±0.97, 1.5±0.66, and 3.2±0.52, respectively. Comparison of the age groups regarding valve leaflets showed that Strut-P1 and Base-P2 were significantly different. Comparison of the valve leaflets and sub-valve indicators between the two genders reflected no significant differences. Age groups differed significantly in terms of Strut-P1 and Base-P2 (P=0.004 and P=00.1, respectively).

**Conclusions:**

A2-P3, A2-P1, anatomic perimeter, and anatomic area were found to be related to gender. A2-P1 and A2-P2 and some leaflet indicators such as Strut-P1 and Base-P2 were associated with age, whereas Base-P2 was affected by body mass index.

## Introduction

Access to detailed knowledge on the dimensions of heart valves, particularly before planning for annuloplasty, is of great importance, which is usually provided by diagnostic procedures [1]. The effectiveness of each test depends on its ability to diagnose disorders with high accuracy. However, appropriate measurement of structural abnormalities should be performed in advance based on normal data; physiological differences such as age, body mass index (BMI), and gender; and factors affecting physiology, namely ethnicity [2]. So far, the normal sizes of heart valves have been investigated considering age, gender, stature, weight, and body surface area; however, they have considered small populations [3]. Data obtained from Western European, American, and Caucasian populations are currently being used as normative reference values for cardiac chamber quantification; however, populations can be varied in this regard. For example, studies performed in Japan indicated different sizes from what has been previously mentioned in the guidelines [4].

There are limited studies on the normal Iranian population determining the normative values for mitral annulus valve dimensions. A recent study has been performed on 368 normal individuals aged between 30 and 70 years; in fact, it was the first study on this issue in Iran. In that study, left ventricular ejection fraction and cardiac chambers were investigated, and it was found that the mean values were lower than what was reported in former guidelines [5].

Therefore, the establishment of population-based guidelines is required to define normative values for cardiac chambers since their application in other populations may be misleading and cause unfavorable clinical decisions. These parameters have not been considered in previous studies, and most studies in this domain have been carried out using echocardiography.

We aimed to perform a morphometric evaluation of the mitral valve since knowledge of the normal dimensions of the valve helps surgeons determine adjustable valves, particularly in annuloplasty where this knowledge is important prior to surgery. Therefore, normative data were determined by measuring the exact dimensions of the mitral valve annulus in healthy and fresh hearts of subjects who had recently died of non-cardiac causes and considering parameters such as age, gender, height, and weight.

## Materials and Methods

In this cross-sectional study, 120 fresh hearts of Iranian patients who had died of non-cardiac causes less than 24 hours before the study were provided for the research team by forensics. First, the hearts were evaluated for their health. The inclusion criteria were healthy hearts of cadavers aged between 25 and 86 years, non-cardiac causes of mortality, and lack of anomalies in the autopsy reports. The exclusion criterion was any anomalies such as mitral valve prolapse or rupture of the lower chords of the valve. Based on this criterion, 32 hearts were excluded from the study. In the next step, BMI (<18.5, underweight; 18.5-24.9, healthy weight; 25-29.9, overweight; and 30-35, obese), age (18-29.9, 30-41.9, 42-53.9, 54-65.9, 66-77.9, and 78-88.9 years), and gender were recorded in the respective forms along with the cause of death and nationality.

Water test was performed on the hearts after washing them with physiological serum. (In this method, the ventricle is filled with water, and if it does not return to the atrium, the valve is considered healthy.)

**Figure 1:**
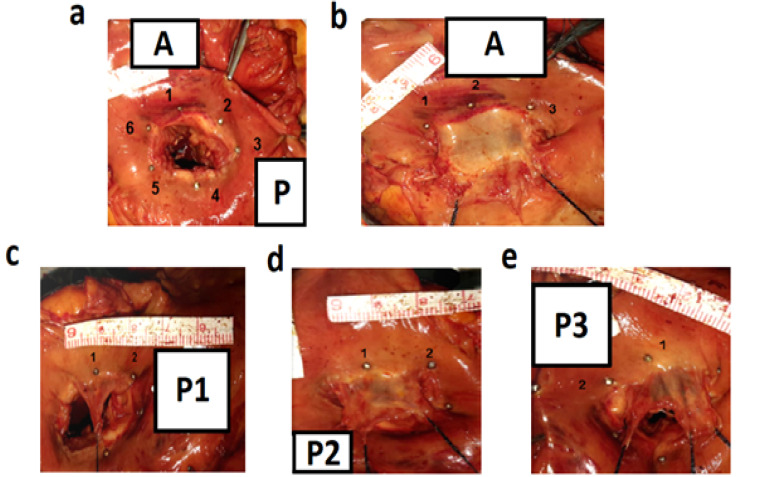
**a**: A, anterior and, P, posterior leaflet of mitral valve; Needle No. 1, commissure A; Needle No. 2, midpoint of anterior leaflet; Needle No. 3, commissure P; Needle No. 4, midpoint of P3 leaflet; Needle No. 5, midpoint of P2 leaflet; Needle No. 6, midpoint of P1 leaflet. **b**: A, anterior mitral valve leaflet; Needle No. 1, commissure A; Needle No. 2, midpoint of the anterior mitral valve leaflet; Needle No. 3, commissure P. **c**: segment P1 from the posterior mitral valve; Needle No. 1, midpoint of segment P1; Needle Number 2, commissure A. **d**: segment P2 from the posterior mitral valve; Needle No. 1, midpoint of the segment P2; Needle No. 2, midpoint of segment P1. **e**: segment P3 from the posterior mitral valve; Needle No. 1, midpoint of segment P3; Needle No. 2, commissure P

The mitral valve annulus was measured using a needle, string, ruler, and imaging as follows: First, the anterior-posterior leaflet was determined and marked at the cleft site with a needle. The posterior leaflet was divided into three segments of P1, P2, and P3; the anterior leaflet was divided into three segments of A1, A2, and A3, which were completely discernible. Accordingly, marking was performed at the commissure between A1-P1 and A3-P3, at the midpoint of A2, and in the middle of P1, P2, and P3 using a needle at the junction of the leaflet with the mitral valve annulus, while a ruler was used at the valve to provide the image of the whole valve (Figure-1).

It should be mentioned that the area between the A1 and P1 is named commissure AL and the area between the A3 and P3 is called commissure PM. In the next step, the edges of the leaflets were loosened and pulled with a suture after the chords were cut. The first image was prepared by locating mark A along with a ruler in the anterior leaflet (Figure-1).

**Figure 2:**
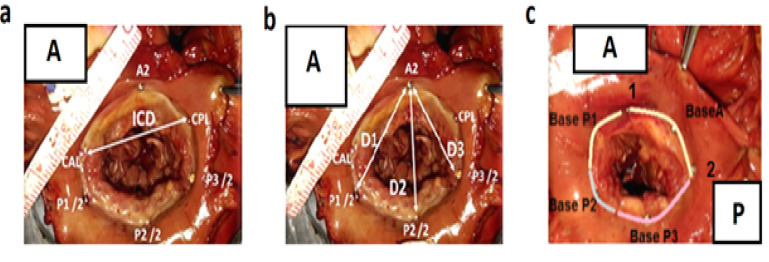
**a**: mitral valve loop; ICD, distance between two commissures A and P; A2, midpoint of the anterior mitral valve; P1/2, midpoint of segment P1; P2/2, midpoint of segment P2; P3/2, midpoint of segment P3. **b**: Mitral valve loop D1, distance between A2 and D2; P1/2, distance between A2 and D3; P2/2, distance between A2 and P3/2. **c**: mitral valve ring; Base A, anterior parts of segments P1, P2, and P3; Needle No. 1, commissure A; Needle No. 2, commissure P

**Figure 3:**
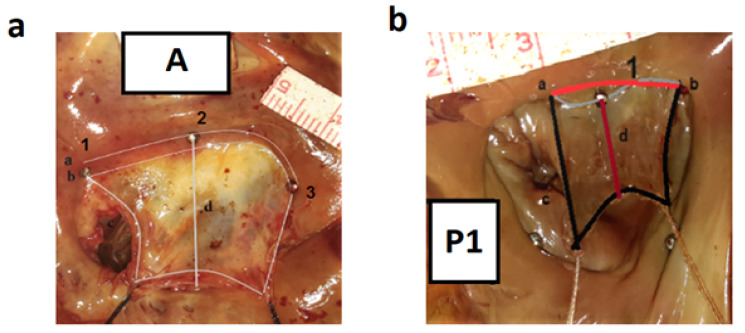
**a**: a, Base A related to the mitral valve loop; b, basal related to the anterior mitral valve leaflet; c, free margin of the anterior mitral valve; d, A2h anterior mitral valve height; Needle No. 1, commissure A; Needle No. 2, midpoint of the anterior mitral valve; Needle No. 3, commissure P. **b**: segment p1 from the posterior mitral valve leaflet; A, base of the mitral valve loop in one segment; B, base of the mitral valve leaflet in one segment; C, free margin or free edge of leaflet in one segment; D, seat height P1 in the posterior part of the mitral valve; Needle No. 1, midpoint of the segment p1

The same steps were repeated for P1, P2, and P3 (Figure-1). Thereafter, a cardiac surgeon familiar with anatomy investigated the valves and quantified the perimeter, length, height, and area of the valves using the ImageJ program. This website is hosted by Laboratory for Optical and Computational Instrumentation at the University of Wisconsin, Madison, USA. Then, anatomic perimeter and area; inter-commissural distance (ICD); A2-P1, A2-P2, and A2-P3 (distance from midpoint of the anterior leaflet [A2] to the three points of P1, P2, and P3 in the posterior leaflet); and Base-P1, Base-P2, Base-P3, and Base-A (the indicators are shown in Figure-2) were calculated. It should be mentioned that Base denotes the base of each leaflet. In the leaflets, we measured the anterior free margin (AFM) surroundings; anterior diameter and area (A2h and Area L); posterior, basal, and marginal areas; marginal to basal ratio (P area [P1 area plus P2 area plus P3 area], basal, marginal, and marginal/basal); and posterior P1, P2, and P3 free margin surroundings, heights, and areas. The number of anterior and posterior papillary muscle heads; tendon heights at A1, P1, A3, and P3 sites (A1, P1, A3, and P3); commissural heights at the anterior and posterior sites (Com AL and PM); number and length of strut chords; and the presence of chords at Base-P1, Base-P2, and Base-P3 were also investigated (Figure-3).

### Statistical Analysis

The obtained data were analyzed using SPSS version 16 (SPSS Inc., Chicago, Illinois, USA). Kolmogorov-Smirnov test was used to assess the normal distribution of the data, while descriptive tests were applied to express frequency and percentage. Chi-square or Fisher exact test was used to compare nominal variables, and independent t-test or its nonparametric equivalent was run to compare the quantitative variables. The one-way analysis of variance or Kruskal-Wallis test was used to compare the quantitative variables among three or more groups. P values less than 0.05 (two-sided) were considered statistically significant.

### Ethical Considerations

Prior to performing the study, we obtained the approval of the Ethics Committee of Mashhad University of Medical Sciences, Mashhad, Iran (Code: ir.mums.fm.rec.1394.370).

## Results

At six months, 88 cadaver hearts were included in this study. Among the study population, 64 (72.7%) patients were men and 24 (27.3%) were women within the age range of 18 to 90 years (Table-1), with a mean height of 169.92±8.879 cm (range: 140-189), mean weight of 69.42±11.220 kg (range, 43-90), and BMI range of 18.5 to 34.9. The major cause of mortality was motor-vehicle accident (28.4%). About 31.8% and 5.7% of the subjects were overweight and obese, respectively.

**Table 1:** Frequencies of Gender, Age, and Cause of Death

		**Number**	**Percent**
**Gender**	Female	64	72.2
	Male	24	27.3
**Cause of death**	Unknown	18	20.5
	Car accident	25	28.4
	Suicide	1	1.1
	Overdose	6	6.8
	Head trauma	1	1.1
	Hanging	5	5.7
	Fall	9	10.2
	Truman	4	4.5
	Poisoning	1	1.1
	Childbirth	2	2.3
	Cancer	2	2.3
	Kill	7	8.0
	Electrocution	1	1.1
	Events	5	5.7
	Down syndrome	1	1.1
**Age groups, y**	18-29.9	20	22.72
	30-41.9	23	26.13
	42-53.9	18	20.45
	54-65.9	16	18.18
	66-77.9	7	7.9
	78.88.9	4	4.54

Normal indicators (anatomic area, anatomic perimeter, ICD, A2-P1, A2-P2, A2-P3, Base-P1, Base-P2, Base-P3, and Base-A), as well as leaflet (AFM, A2h, Area L, P area, Basal, Marginal/Basal ratio, P1FM, P1FM, P1h, P1 area, P2FM, P2 h, P2 area, P3FM, P3 h, and P3 area) and sub-valve area indicators (APM, A1, P1, Com A, Strut No. APM, Strut APM L, Strut P1, Strut P1 L, PPM head, A3, P3, Com P, Strut No. PPM, Strut L PPM, Strut P3, Strut P3 L, Basal P1, Basal P2 and Basal P3) associated with the mitral valve of the Iranian population are presented in Table-2.

**Table 2:** Normal Data of The Investigated Indicators

**Indicator**	**Minimum**	**Maximum**	**Mean±SD**
**Anatomic area (cm)**	2.12	8.7	5.14±1.28
**Anatomic perimeter (Cm** ^2^ **)**	5.77	10.76	8.3±1
**IC (cm)**	1.79	3.82	2.7±0.42
**A2-P1 (cm)**	1.2	3.2	2.27±0.37
**A2-P2 (cm)**	1.1	3.7	2.3±0.43
**A2-P3 (cm)**	1.2	3.1	2.06±0.35
**Base-P1 (cm)**	0.89	3.1	1.66±0.43
**Base-P2 (cm)**	0.52	9.3	1.2±0.97
**Base-P3 (cm)**	0	5.47	1.5±0.66
**AFMMit**	3.131	9.46	5.23±0.96
**A2H**	1.359	3.25	2.28±0.37
**AreaL**	2.08	9.405	5±1.27
**PArea**	1.508	9.217	4.52±1.31
**Basal**	0.335	9.4	0.99±0.97
**Marginal**	0.824	2.095	1.4±0.3
**Marginal Basal**	0.6	4.5	1.76±0.72
**P1FM**	1.326	5.395	2.91±0.86
**P1H**	0.715	1.864	1.26±0.26
**P1Area**	0.468	3.948	1.28±0.54
**P1FMType**	1	5	2.76±1.63
**P2H**	0.844	2.033	1.34±0.27
**P2Area**	0.426	4.365	1.97±0.73
**P2FMType**	1	6	3.01±1.32
**P2FM**	1.463	6.342	3.63±0.98
**P3FM**	1.053	6.579	3.09±1.07
**P3H**	0.606	1.903	1.15±0.27
**P3Area**	0.284	6.764	1.34±0.87
**P3FMType**	1	5	2.91±1.45
**APMHeadNo**	1	5	3.18±0.63
**A1**	1	2.5	1.8±0.34
**P1**	1	2	1.57±0.29
**ComA**	0.5	5	1.42±0.53
**StratA**	1	2	1.02±0.15
**StratANo**	1	6	2±0.93
**StratL**	1	3.5	2.21±0.48
**StratP1**	1	2	1.52±0.5
**StratP1No**	0	6	1.83±1.29
**StratP1L**	0	2.9	1.81±0.59
**PPMHeadNo**	2	8	3.28±0.93

In the our study, indicators of A2-P3, A2-P1, anatomic perimeter, and anatomic area showed significant differences between men and women (Table-3). Indicators of A2-P2 and A2-P1 were significantly different across various age groups (P=0.05; Table-4). Base-P2 indicator exhibited significant differences in various BMI groups (P<0.05).

**Table 3:** Significant Differences in the Parameters Based on the Gender of the Subjects. All Data are Presented as mean±SD

**Indicators**	**Male**	**Female**	**P-value** ^*^
**Anatomic area (cm)**	5.55±1.26	4.71±1.19	0.039
**Anatomic perimeter (cm** ^2^ **)**	8.67±0.9	8±1.06	0.031
**A2-P1 (cm)**	2.37±0.35	2.15±0.34	0.041
**A2-P3 (cm)**	2.15±0.35	1.91±0.36	0.031

* Mann-Whitney U test

**Table 4:** Mean Distribution of Parameters A2-P1 and A2-P2 Among the Study Population

**Age groups**	**A2-P1** **(Mean±SD)**	**A2-P2** **(Mean±SD)**
**18-29.9 years (cm)**	2.01±0.35	2.01±0.39
**30-41.9 years (cm)**	2.45±0.39	2.47±0.5
**42-53.9 years (cm)**	2.39±0.37	2.61±0.42
**54-65.9 years (cm)**	2.37±0.27	2.41±0.36
**66-77.9 years (cm)**	2.22±0.15	2.40±0.26
**78-89.9 years (cm)**	2.30±0.31	2.29±0.22
**P-value** ^*^	0.024	0.007

* Kruskal-Wallis

Considering the mitral valve leaflets, posterior free margin (P1-FM) types were not significantly different among the subjects (P=0.4). In the comparison of P2-FM and P3-FM types, the presence of strut anterior (Strut A), strut posterior No. 1 (Strut P1), strut posterior No. 2, strut posterior No. 3, Base-P1, Base-P2, and Base-P3 and BMI were not significantly different (P=0.14, P=0.26 0.93, P=0.084, P=0.73, P=0.13, P=0.92, P=0.86, and P=0.47, respectively).

Comparison of the valve leaflets in the two genders reflected that P1-FM, P2-FM, and P3-FM types, as well as P2-P3, were not significantly different (P=0.4, P=0.46, P=0.7, and P=0.42, respectively). In the sub-valve area, Strut-A, Strut-P1, Strut-P2, Strut-P3, Base-P1, Base-P2, and Base-P3 were not significantly different between the two genders (P=0.51, P=0.84, P=0.45, P=0.61, P=0.3, P=0.09, and P=0.49, respectively).

Comparison of age groups in terms of valve leaflets demonstrated that Strut-P1 and Base-P2 were significantly different in various age groups (P=0.004 and P=00.1, respectively). However, P1-FM, P2-FM, and P3-FM types, P2-P3, and Strut-A were not significantly different between the two genders (P=0.91, P=0.55, P=0.9, P=0.5, and P=0.86, respectively). In the sub-valve area, Strut-A, Strut-P2, Strut-P3 Base-P1, and Base-P3 were not significantly different among various age groups (P=0.68, P=0.39, P=0.1, P=0.31, and P=0.26, respectively).

## Discussion

The present study focused on the mitral valve, on which there are limited studies. We considered the effects of gender, height, weight, and ethnicity on valve dimensions, while previous studies have seldom taken into account these indicators, and most studies were performed using echocardiography.

Our study is more comprehensive than the previous ones since it considers the effects of age, gender, height, and weight on valve dimensions in normal Iranian population. Most previous studies were conducted using echocardiography, while we performed this study on fresh hearts and considered almost all the potential aspects.

Kopui *et al*., (1995) investigated 30 formalin-fixed hearts. To determine the shape of the fibrous ring of the mitral valve, we sectioned the hearts and performed morphometric, radiographic, and histological methods to study them. The fibrous ring adopted a horizontal “S” shape around the two commissures, and the average distance between the highest and lowest parts of the fibrous ring was measured to be 7.1 mm (range, 6.2-12.5). Radiographic evaluation revealed that the fibrous ring lies approximately in two planes rather than in one. The histological study showed that the fibrous tissue of the mitral valve is continuous with the aortic fibrous ring and that a slight but significant thickening marks the insertion of the mitral valve.

Thus, the mitral valve lies in two planes, and it seems that changes in the fibrous ring may influence valve perimeter and area; this information can be beneficial for cardiac surgery and valvuloplasty [6].

Masataka *et al*. (1013) found that a segment of the annular fibrous structure was always facing directly toward the left ventricular cavity; the length of the segment ranged from 1.0 to 3.4 mm. With regard to aortic annulus size, there were large variations within and among subjects. The shortest distance between the mitral annulus and the left circumflex artery was at the anterolateral commissure (3.3 mm).

It seems that age and BMI can influence the fibrous structure and, in turn, perimeter and area of the valve [7].

Girish *et al*. (2014) found that the dimensions of the mitral valve annulus were larger in men than in women and greater at end-diastole than end-systole. In the present study, indicators were greater in men compared with women, and it seems that these results are somehow in line with those of studies performed using echocardiography [8].

With advancing age, Keller *et al.* (1999) revealed a rise in the number of fibers per measuring area in both genders. We noted that through the effects of age and gender, fibrous tissues play a role in mitral valve changes [9].

In the study by Sonne *et al*, mitral valve annular area showed the highest correlation with body surface area, followed by interpapillary distance and posteromedial (PM) and anterolateral (AL) papillary muscle distance. Age was not associated with either annular or tenting parameters, but it had a moderate negative correlation with interpapillary muscle angle and a mild negative correlation with interpapillary distance, both normalized by body surface area. It seems that changes in the perimeter and area of the valve were due to alterations in papillary muscles [10].

In a study performed in Iran, Mohammadi *et al*., examined cardiac size in a cadaveric study and reported that the mean values for the heart length and width were similar to those of previous reports from Western countries. They presented that the circumference of the tricuspid valve was less than what has been previously established in the literature, while circumference of the mitral valve was higher than that. In line with our study, Mohammadi *et al.* considered the valves and their muscles in general with few details [11].

## Conclusion

We found that gender affects A2-P3, A2-P1, anatomic perimeter, and anatomic area of the mitral valve and that the size of indicators was larger in men compared to women. We found that A2-P1 indicator had the largest size in the 30 to 41.9 age group; nevertheless, it slightly decreases with advancing age, and A2-P2 indicator starts to grow from the first age group to reach to its maximum in the third age group, after which it dwindles significantly. Investigations showed that Base-P2 decreases significantly with increased BMI. Moreover, in leaflet and sub-valve area, some leaflet indicators such as Strut-P1 and Base-P2 were associated with age, while Base-P2 was affected by BMI.

## Conflict of Interest

None to declare.
